# Static and dynamic mechanics of the murine lung after intratracheal bleomycin

**DOI:** 10.1186/1471-2466-11-33

**Published:** 2011-05-31

**Authors:** Effrosyni D Manali, Charalampos Moschos, Christina Triantafillidou, Anastasia Kotanidou, Ioannis Psallidas, Sophia P Karabela, Charis Roussos, Spyridon Papiris, Apostolos Armaganidis, Georgios T Stathopoulos, Nikolaos A Maniatis

**Affiliations:** 1 2nd Pulmonary Department, "Attikon" General Hospital, National and Kapodistrian University of Athens Medical School, Haidari, Greece; 2 Applied Biomedical Research & Training Center "Marianthi Simou", 1st Dept. of Critical Care & Pulmonary Services, "Evangelismos" General Hospital, National and Kapodistrian University of Athens Medical School, Athens, Greece; 3 1st Dept. of Critical Care & Pulmonary Services, "Evangelismos" General Hospital, National and Kapodistrian University of Athens Medical School, Athens, Greece; 4 2nd Dept. of Critical Care, "Attikon" General Hospital, National and Kapodistrian University of Athens Medical School, Haidari, Greece

## Abstract

**Background:**

Despite its widespread use in pulmonary fibrosis research, the bleomycin mouse model has not been thoroughly validated from a pulmonary functional standpoint using new technologies. Purpose of this study was to systematically assess the functional alterations induced in murine lungs by fibrogenic agent bleomycin and to compare the forced oscillation technique with quasi-static pressure-volume curves in mice following bleomycin exposure.

**Methods:**

Single intratracheal injections of saline (50 μL) or bleomycin (2 mg/Kg in 50 μL saline) were administered to C57BL/6 (*n *= 40) and Balb/c (*n *= 32) mice. Injury/fibrosis score, tissue volume density (TVD), collagen content, airway resistance (*R_N_*), tissue damping (*G*) and elastance coefficient (*H*), hysteresivity (*η*), and area of pressure-volume curve (PV-A) were determined after 7 and 21 days (inflammation and fibrosis stage, respectively). Statistical hypothesis testing was performed using one-way ANOVA with LSD *post hoc *tests.

**Results:**

Both C57BL/6 and Balb/c mice developed weight loss and lung inflammation after bleomycin. However, only C57BL/6 mice displayed cachexia and fibrosis, evidenced by increased fibrosis score, TVD, and collagen. At day 7, PV-A increased significantly and *G *and *H *non-significantly in bleomycin-exposed C57BL/6 mice compared to saline controls and further increase in all parameters was documented at day 21. *G *and *H*, but not PV-A, correlated well with the presence of fibrosis based on histology, TVD and collagen. In Balb/c mice, no change in collagen content, histology score, TVD, *H *and *G *was noted following bleomycin exposure, yet PV-A increased significantly compared to saline controls.

**Conclusions:**

Lung dysfunction in the bleomycin model is more pronounced during the fibrosis stage rather than the inflammation stage. Forced oscillation mechanics are accurate indicators of experimental bleomycin-induced lung fibrosis. Quasi-static PV-curves may be more sensitive than forced oscillations at detecting inflammation and fibrosis.

## Background

Pulmonary fibrosis (PF), the progressive destruction of functional lung parenchyma and its conversion to scar tissue, can lead to severe decline in lung function, dyspnoea and death [1-3]. Among the various methods available to model PF in laboratory animals, the bleomycin model is the most widely used. Systemic or local pulmonary administration of bleomycin elicits an initial inflammatory reaction, followed by fibrosis at later time-points [4-7]. Despite its extensive use, little is known on the magnitude and time-course of lung functional alterations, which take place in this type of injury, owing partly to the technical difficulty of measuring lung function in mice. In addition to gaining important pathophysiological insight on the effects of collagen deposition in the lung, precise knowledge of lung function would be important in order to quantify the severity of physiological impairment imposed on the animals, as well as to measure treatment responses.

Quantification of lung fibrosis induced by bleomycin is challenging, largely relying on histology and determination of lung collagen content. Both methods, however, suffer from limitations, including user-dependency and stringent technical requirements [4,6-12]. Thus, objective assessment of lung dysfunction would be of benefit. Reports of lung function measurements in this model have yielded conflicting results. Using the technically challenging and highly invasive alveolar capsule technique in open-chested rats, Dolhnikoff *et al*. observed increased lung elastance in bleomycin-exposed animals, which correlated with collagen content of lung tissue sections but not with α-smooth muscle actin or elastin [8]. Conversely, in the study by Goldstein *et al*., lung collagen or elastin did not correlate with lung mechanics, as assessed by quasi-static Pressure-Volume (PV) curves [13]. Although collagen accumulation and histologic alterations imply the presence of lung dysfunction, the corresponding mechanical impairment of the respiratory system, a major determinant of the severity of the experimental intervention, cannot be precisely quantified, unless some lung function measurement has been conducted.

Highly sensitive airway pressure transducers developed during the past fifteen years have enabled objective lung function determinations on live mice and rats [8,14-16]. The forced-oscillation technique (FOT), a dynamic measurement of lung impedance to airflow, is based on the observation that lung elastance and airway resistance vary within the physiological respiratory frequency range [17,18]. To accurately describe lung mechanical properties, pulmonary visco-elastic behaviour is assessed over a range of respiratory frequencies. Using a mathematical tool known as the constant phase model, lung impedance measurements obtained in the above fashion can be broken down to components attributable to airway resistance *R_N _*and inertance *I *in addition to lung tissue elastance *H *and tissue damping or energy dissipation *G *[17]. With technical advancements, the method became available for mouse studies and is expected to offer the greatest degree of accuracy and specificity, not only since *in vivo *lung function determinations are carried out under precise experimental control [8,14,15], but also since lung mechanical properties are described by a set of four variables encompassing a wide spectrum of respiratory frequencies. Using this methodology, Cohen *et al*. were able to elucidate previously unrecognized lung dysfunction in cystic fibrosis trans-membrane conductance regulator-deficient (*cftr*^-/-^) mice, [19] while other groups have used it to quantify lung pathology in asthma [20,21]. However, the advantage of the technique over the more widely available measurement of lung compliance using quasi-static PV-curves has not been experimentally demonstrated. In addition, changes in lung mechanics after bleomycin delivery to mice are incompletely understood.

The present study addresses lung structure-function relationships in the bleomycin model. To this end, we used two different methodologies to describe the temporal aspects of lung functional derangements and compared these to established biochemical and morphological indices of lung pathology. To induce fibrosis, bleomycin was delivered intratracheally (*it*) to C57BL/6 mice, which were sacrificed after seven (early time-point; predominance of inflammatory changes) or 21 days (late time-point; predominance of fibrotic changes). Saline-treated C57BL/6 mice as well as Balb/c mice, which are known for their resistance to bleomycin [7,22-24], served as negative controls. Lung functional parameters obtained at both time-points were complemented by biochemical and morphological assays, in order to characterize the different phases of the model from a structural and functional perspective.

## Methods

### Mouse Model

Experiments were conducted according to international standards [25], and were approved by the by Veterinary Administration Bureau, Prefecture of Athens, Greece (Ms. C. Kapetanopoulou and A. Tsigarida, Protocol #K/3459). Male 8-12 week-old C57BL/6 and Balb/c mice (Hellenic Pasteur Institute, Athens, Greece) received *it *saline (50 μL) or bleomycin (Nippon Kayaku, Tokyo, Japan; 2 mg/Kg in 50 μL saline), as previously described [6,26]. Mice were killed by intraperitoneal (*ip*) pentothal sodium overdose at early (day 7) or late time-points (day 21) for determination of lung injury and fibrosis, respectively. Only a subset of mice was subjected to lung function measurements prior to sacrifice; this was done in order to determine whether lung function measurements *per se *alter biochemical and morphological readouts of bleomycin-induced lung fibrosis. Thereafter, the right main stem bronchus was ligated, the right lung was frozen in liquid N_2 _for collagen content measurement and the left lung was inflated with neutral-buffered formalin at 20 cm H_2_O for tissue staining procedures.

### Lung function

Following anaesthesia (75 mg/Kg *ip *pentothal sodium), placement of tracheostomy and muscle paralysis (8 mg/Kg *ip *succinyl choline), mice were connected to a Flexivent rodent ventilator (Scireq, Montreal, Ontario, Canada). Prior to measurements, the volume history of the lung was standardized with two 6-second deep inflations to a pressure limit of 30 cm H_2_O. After an initial 3-min run-in period of ventilation with FiO_2 _= 0.21, tidal volume 8 mL/Kg, respiratory rate 150 breaths/min, and positive end-expiratory pressure (PEEP) = 0 cmH_2_O, we obtained total respiratory system impedance by applying an 8-second pseudorandom frequency oscillation (0.5-19.75 Hz) to the airway opening. Measurements were performed 3 times at 30 sec intervals. Data were fit into the constant phase model in order to fractionate total respiratory input impedance into airways resistance (*R_N_*), tissue damping coefficient (*G*) and tissue elastance coefficient (*H*) [15,27]. Hysteresivity (*η*) was calculated as the ratio of G to H. The procedure lasted 15 minutes.

To obtain PV curves, the lung was incrementally inflated to 30 cmH_2_O and airway pressures were recorded on each volume increment. Area of PV curve-(PV-A) was calculated by the Flexivent software and the slope of the linear portion of the in-and expiratory limb of the curve was manually calculated.

### Histology

Lungs were fixed in formalin (24 hours) and maintained in 70% ethanol for 3 days, when they were embedded in paraffin. Five μm-thick sagittal sections were cut and subsequently stained with Hematoxylin/Eosin and trichrome blue [6]. Ten sequential, non-overlapping fields (Å = 200) from each lung were evaluated by two blinded readers (EDM, GTS) for inflammation/injury (0, no inflammation; 1, focal interstitial infiltrates; 2, diffuse interstitial infiltrates; 3, focal alveolar infiltrates; 4, confluent alveolar infiltrates/consolidation) and fibrosis (0, normal lung; 1, thickening of <50% of interalveolar septa; 2, thickening of ≥50% of interalveolar septa; 3, interalveolar septal thickening with formation of isolated fibrotic foci; 4, multiple fibrotic foci with distortion of parenchymal architecture) using 0-4 point scales. Tissue volume density (TVD), the percentage of lung volume occupied by tissue, was determined using analysis of ten sequential, non-overlapping fields (Å = 200) on tissue sections from each lung by ImageJ freeware (Rasband 1997-2008, available at http://rsb.info.nih.gov/ij. Briefly, colour digital images were obtained, converted to binary images, and the percentage of tissue pixels to whole lung pixels was calculated. We did not measure a reference volume; instead, we expressed the proportion of lung volume occupied by tissue as a percentage and not as an absolute number.

### Collagen determination

The Sircol collagen assay (Biocolor, Belfast, Ireland) was used to measure soluble collagen [28] in whole-lung protein extracts performed as described elsewhere [6].

### Statistical analysis

All values represent mean ± Standard Deviation. One-way ANOVA with LSD post-hoc tests were used to compare means. Associations were assessed using Pearson's correlation. All *P *values are two-tailed; *P *values < 0.05 were considered significant. *P*-values less than 0.05 are marked by one asterisk; *P*-values less than 0.01 by two asterisks, and *P*-values less than 0.001 by three asterisks. Statistical analyses were done using Statistical Package for the Social Sciences v.13.0.0 (Chicago, IL)

## Results

### Body weight in response to bleomycin

**Figure 1:**
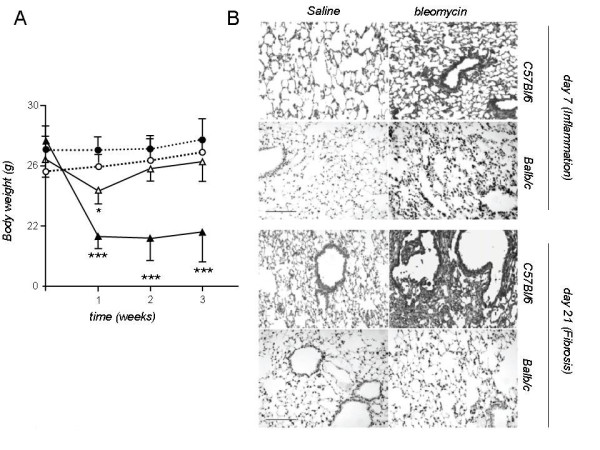
**Changes in body mass and lung microstructure after intratracheal bleomycin to a sensitive (C57BL/6) and a resistant (Balb/c) mouse strain**. C57BL/6 (*n *= 38) and Balb/c (*n *= 32) mice received single doses of 2 mg/kg intratracheal bleomycin or saline control (injection volume = 50 μl). **A**. Both C57BL/6and Balb/c developed early weight loss after bleomycin (but not saline). Only C57BL/6mice displayed persistent cachexia after bleomycin. **B**. Representative histologic images of lungs at early (7 days after intratracheal bleomycin), and late (21 days after intratracheal bleomycin, respectively) time-points. (Scale bar = 200 μm). (***P < .001 compared with C57BL/6 N/S; *P < .05 compared with Balb/c N/S). *Data points*, mean; *bars*, SD. Solid Circles: C57BL/6-Saline; Open circles: Balb/c-saline; Solid triangles: C57BL/6-bleomycin; Open triangles: Balb/c-bleomycin).

We initially sought to recapitulate the reported susceptibility of C57BL/6 mice to pulmonary fibrosis induced by *it *bleomycin [4,5,7,22,24-26,29-31] using established methods. We therefore compared the C57BL/6 strain against Balb/c mice, known to display minimal lung fibrosis in response to bleomycin, as well as to mice that received saline as internal controls. The number and group allocation of mice utilized for the present studies are shown in Table 1. Both C57BL/6 and Balb/c mice developed significant weight loss after *it *bleomycin, but not after saline control (Figure 1). In fibrosis-resistant Balb/c mice, body weight decreased initially, reaching its nadir one week after injection and recovering by week 2 (Figure 1). In the fibrosis-prone C57BL/6 strain, weight loss ensued early and recovery did not occur within the observation period (Figure 1).

**Table 1:** Sample size and group allocations.

		*early harvest* *(day 7)*	*late harvest* *(day 21)*
**C57BL/6**	*saline*	8(4)	8(4)
	*bleomycin*	13(10)	11(7)

**Balb/c**	*saline*	7(5)	8(8)
	*bleomycin*	8(5)	9(9)

Number, strain and treatment of mice utilized for the present studies, given as total number (number of mice with lung mechanics measurements).

### Histologic and biochemical indices of inflammation and fibrosis

**Figure 2:**
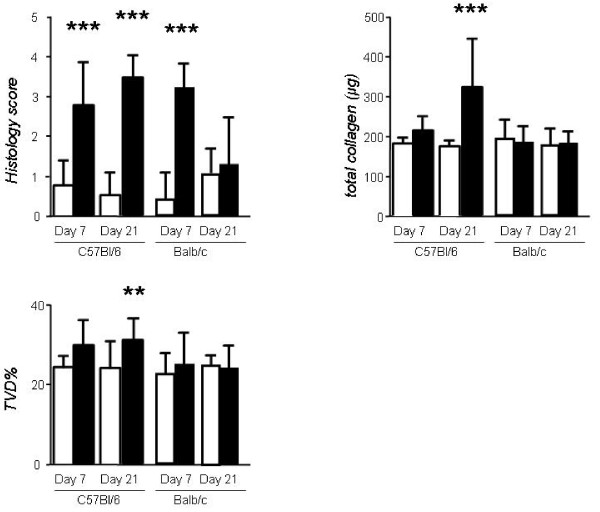
**Established parameters of mouse bleomycin-induced lung injury and fibrosis: lung histological score, collagen and tissue volume density**. Mice were treated with bleomycin (black columns) or saline (white columns) as in Fig. 1 and were sacrificed at early and late time-points. Lungs were sectioned for histologic analyses (left lungs) or homogenized for collagen measurement (right lungs). *P < 0.05, **P < 0.01, and ***P < 0.001 compared with saline-treated controls. *Columns*, mean; *bars*, SD. TVD, tissue volume density.

The acute inflammatory response to bleomycin was evidenced by marked alterations in pulmonary microanatomy, including alveolar septal thickening and infiltration of the parenchyma by inflammatory cells, resulting in increased lung injury scores (Figures 1, 2), but not in increased TVD and lung collagen content at day 7 (Figure 2). This response was of similar magnitude in both strains. In contrast to Balb/c mice, C57BL/6 mice proceeded to lung fibrosis development following bleomycin, but not saline (Figures 1, 2). The fibrotic response of C57BL/6 mice to bleomycin was mirrored by significantly increased lung fibrosis score, TVD, and lung collagen content at day 21 (Figure 2). Histologically, the observed collagen distribution pattern was primarily bronchocentric, in agreement with previous reports [8].

### Lung function testing does not interfere with readouts of fibrosis

We next sought to determine whether measuring lung function *in vivo *affects histologic-and collagen-assisted evaluation of lung fibrosis. To this end, we compared classic measures of bleomycin-induced lung fibrosis (fibrosis score of lung tissue sections, TVD, and lung collagen) between mice that did or did not undergo lung function testing prior to sacrifice. We found that *pre mortem *lung function determinations had no impact on any of the above parameters. (Table 2). These results indicate that lung mechanics measurements prior to sacrificing experimental mice do not alter the results obtained by lung histology and collagen determinations.

**Table 2:** Established end-points of bleomycin-induced lung injury and fibrosis in mice that did or did not undergo lung function measurements.

	*day 7*		*day 21*	
	***C57BL/6***	***Balb/c***	***C57BL/6***	***Balb/c***

***no lung function measurements performed***				
*n*	3	3	4	0
*lung injury score*	2.8 ± .5	3.0 ± .0	3.8 ± .8	-
*TVD (%)*	30.5 ± .2	26.5 ± 7.4	30.3 ± 3.4	-
*lung collagen (μg)*	205 ± 99	185 ± 92	320 ± 230	-
***lung function measurements performed***				
*n*	8	5	7	9
*lung injury score*	2.8 ± 1.7	3.4 ± 0.4	3.6 ± .5	1.3 ± 1.5
*TVD (%)*	29.5 ± 9.6	23.7 ± 6.5	31.5 ± 3.4	24.3 ± 6.3
*lung collagen (μg)*	211 ± 102	177 ± 101	325 ± 312	179 ± 69

Data are presented as mean ± SD. No significant differences were observed. TVD: Tissue volume density.

### Lung functional alterations induced by bleomycin using forced-oscillation lung mechanics

We subsequently evaluated the time course of lung mechanical alterations using the constant-phase model and examined how these correlate with morphological/biochemical markers of acute inflammation and fibrosis. We therefore mechanically ventilated the majority of the mice enrolled in these studies prior to sacrifice (Table 1). Lung function determinations in control and bleomycin-treated C57BL/6 and Balb/c mice lasted roughly fifteen minutes per mouse.

**Figure 3:**
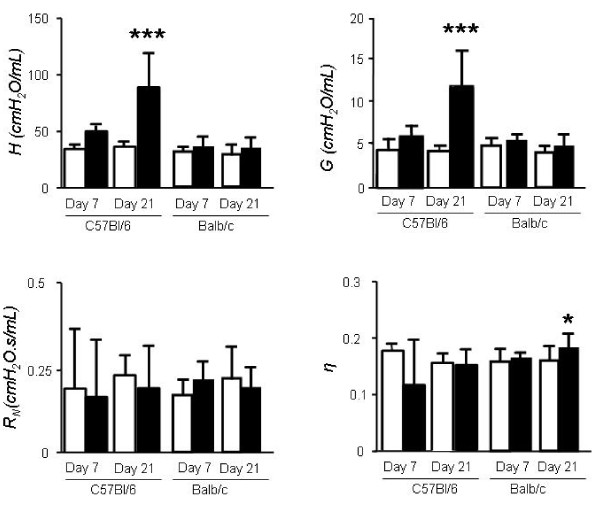
**Mechanical parameters of lung injury and fibrosis induced by bleomycin administration to resistant (Balb/c) and sensitive (C57BL/6) mouse strains**. Mice were treated with bleomycin (black columns) or saline (white columns) as in Fig. 1 and lung function was determined using the forced oscillation technique and the constant phase model at early and late time-points. *P < 0.05, **P < 0.01, and ***P < 0.001 compared with saline-treated controls. *Columns*, mean; *bars*, SD. *R_N_*, airway resistance; *H*, lung tissue elastance; *G*, lung tissue damping; *η*, hysteresivity.

In the early inflammatory phase of bleomycin-induced lung injury (day 7) in C57BL/6 mice, we found modest, non-significant increases in *G *and *H *(Figure 3). Interestingly, in Balb/c mice, despite clear histological evidence of inflammation, *G *and *H *were no different from saline controls.

On day 21 post-bleomycin, *G *and *H *were increased significantly in the lungs of C57BL/6 mice compared to saline controls (Figure 3), in temporal relationship to the development of fibrosis. Notably, the rise in *G *and *H *was more pronounced in the late time point (fibrosis) compared to the early time point. Specifically, *G *increased by 97% from day 7 to day 21 (p < 0.05) while *H *rose by 78% (p < 0.05, Figure, 4). No significant changes were induced by bleomycin on *R_N_*and *η *on any of the strains.

**Figure 4:**
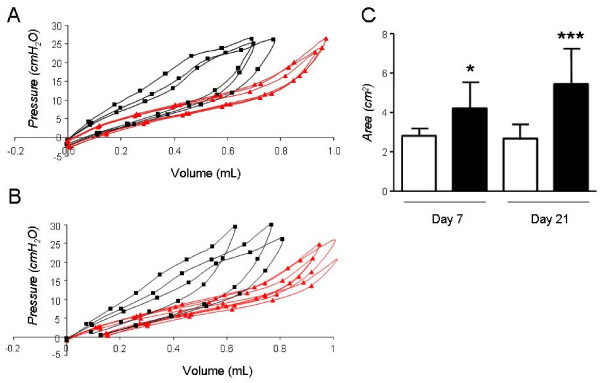
**Pressure-Volume curves in C57BL/6 mice**. Sample Pressure-Volume curves obtained 7 days (A) and 21 days (B) after intratracheal bleomycin (black squares) or saline (grey triangles) to C57BL/6 mice. Cumulative results of PV-curve area for early and late time-points.(**C**). *P < 0.05, **P < 0.01, and ***P < 0.001 compared with saline-treated controls. *Columns*, mean; *bars*, SD.

### Lung functional alterations induced by bleomycin using pressure-volume curves

**Figure 5:**
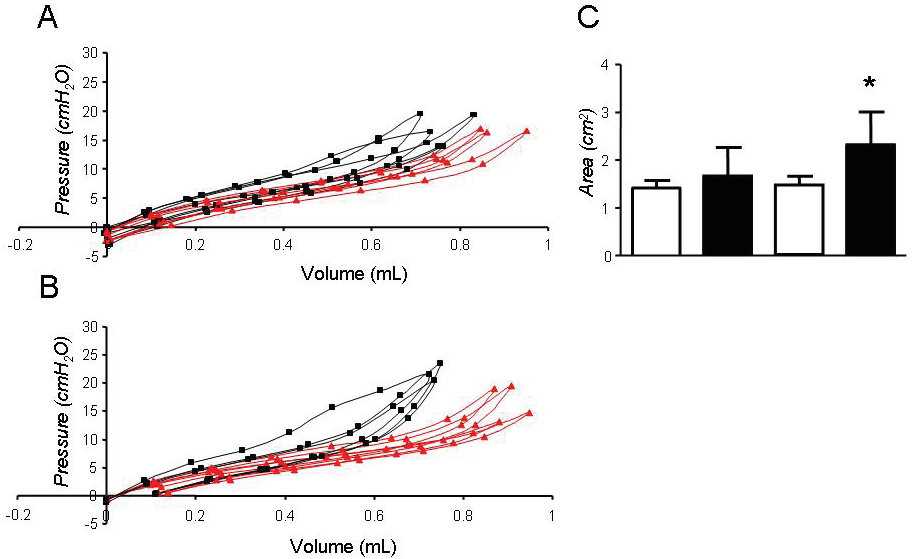
**Pressure-Volume curves in Balb/c mice**. Sample Pressure-Volume curves obtained 7 days (**A**) and 21 days (**B**) after intratracheal bleomycin (black squares) or saline (gray triangles) to Balb/c mice. Cumulative results of PV-curve area for early and late time-points (**C**). *P < 0.05 compared with saline-treated controls. *Columns*, mean; *bars*, SD.

We undertook a comparison of lung mechanics measured by FOT to the more traditional method of quasi-static PV-curves. We specifically looked at the area enclosed within the PV-curve (PV-A), a measure of lung hysteresis, in addition to the slope of the in-and expiratory limb of the PV-curve. In C57BL/6 mice, bleomycin led to an upward and leftward shift of the in-and expiratory limb of the PV curve as early as day 7; these changes were associated with increases in PV-A (Figure 4) in addition to the slopes of the in-and expiratory limb of the PV curve (not shown). PV-A and slopes increased further at day 21, although to a lesser extent compared to the rise in *G *and *H*. Specifically, PV-A increased from day 7 to day 21 by 29.3% (Figure 4). Surprisingly, in Balb/c mice, abnormal PV curve morphology was noted at day 7, although the increase in PV-A was non-significant. After 21 days however, PV-A and slopes increased significantly in the bleomycin group compared to saline controls and these changes were not detected by the FOT (Figure 5).

### Lung function testing by FOT detects the presence of fibrosis by intratracheal bleomycin

**Figure 6:**
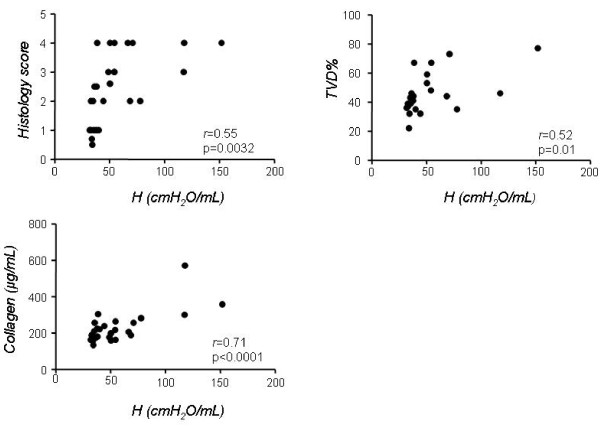
**Correlation of lung elastance by forced oscillation method with morphological and biochemical indices in the bleomycin mouse model**. Correlation of lung elastance H with histology score, tissue volume density (TVD) and lung collagen in all C57BL/6mice irrespective of group allocation. r = correlation coefficient.

**Figure 7:**
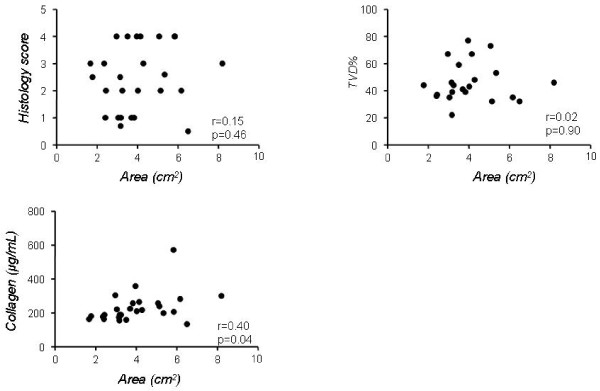
**Correlation of pressure-volume curves with morphological and biochemical indices in the bleomycin mouse model**. Correlation of lung PV-curve area with histology score, tissue volume density (TVD) and lung collagen in all C57BL/6mice irrespective of group allocation. r = correlation coefficient.

To investigate which of the two different methodologies tested more clearly describes the morphological and biochemical alterations of the two phases of the bleomycin model, we correlated values of *G*, *H *and PV-A with histological score, lung collagen and TVD in C57/B6 mice (Figures 6 and 7). To this end, we plotted the values of *G*, *H *and PV-A of each mouse, irrespective of its group allocation, against the respective values for histological score, lung collagen and TVD obtained on the same animal. We observed that *H *values correlated well with the presence of fibrosis as determined by the classical parameters. In particular, the correlation between *H *and collagen was the most striking (*r *= 0.71, p < 0.0001), while the correlation to histological score and TVD, although still highly significant, was not as close (*r *= 0.55 and 0.52 respectively, Figure 6). Similar correlation data were obtained for *G *(*r *= 0.48 vs. histological score, *r *= 0.52 vs. TVD and *r *= 0.68 vs. collagen, p < 0.05 for all three, not shown).

In contrast to the data obtained for FOT parameters, PV-A values correlated only poorly with fibrosis indices. The correlation coefficient *r *vs. histological score and TVD was 0.15 and-0.03 respectively (p > 0.05 for both), whereas PV-A weekly correlated to collagen ratio (r = 0.4, p = 0.04, Figure 8).

## Discussion

We undertook a systematic survey of lung mechanical behaviour in the bleomycin model in order to describe the time-course of lung functional alterations and establish relationships between structural and functional parameters of lung injury and fibrosis. We used local pulmonary delivery of bleomycin to sensitive (C57BL/6) and resistant (Balb/c) mice. Negative controls, which received saline, were enrolled in each study arm. Pulmonary function determinations using the forced oscillation technique and PV curve analysis were easy and rapid and did not compromise subsequent biochemical assays. In fibrosis-prone C57BL/6 mice, bleomycin exposure led to an early inflammatory response characterized by a modest increase in lung elastance. The establishment of fibrosis led to a further, substantial increase in lung stiffness. The FOT more specifically detected the presence of fibrosis, while quasi-static PV-curves were more sensitive indicators of lung dysfunction in global terms, yielding pathological values in C57BL/6 mice at the early (inflammation) time-point as well as in Balb/c mice.

Pulmonary fibrosis, regardless of whether idiopathic or occurring in the setting of systemic disease [1], leads to destruction of lung architecture and lung functional decline. Among the various predictors of outcome studied, serial lung function testing is so far the most useful predictor of death [32]. It would thus appear that, for a bleomycin experiment to adequately model human fibrosis, some degree of functional impairment should be present. Yet few studies have investigated functional alterations in response to bleomycin in mice [9,33-35] and only a small minority of bleomycin studies report lung function results, possibly due to the required technical expertise and cost. Thus, despite their substantial drawbacks lung histology and collagen content determinations remain the established means of assessing bleomycin-induced dysfunction. Histological measurements are not only subject to observer bias and sampling errors, but also require the application of expensive and/or time-consuming processing, such as morphometry [36]. Collagen measurement, the gold standard for evaluating murine lung fibrosis, does not give spatial information [37] and, so far, has not been clearly shown to correlate with lung mechanics [4,8,13]. In addition, the amount of collagen may not be the sole determinant of lung mechanical properties; rather, the spatial organization of collagen fibres and the degree of cross-linkage as well as connections to other matrix components may be critical [38,39]. Importantly, treatment interventions may have a positive impact on lung dysfunction in the bleomycin model without changing collagen content or histology [9]. Finally, even if collagen deposition and histological alterations consistent with fibrosis can be detected, their functional importance cannot be assessed without lung function testing.

The FOT is gradually emerging as an objective and detailed means of assessing lung function in normal and diseased lungs. Because lung tissue elastance and airway resistance vary with respiratory rate [27], measuring frequency-dependent total lung impedance and breaking it down to its resistive and elastic components may provide physiological information, which would be missed if only static PV-curves were to be determined [40]. By fitting frequency-dependent lung impedance data into the constant-phase model, lung mechanical properties can be described using four parameters: airway resistance *R_N_*, inertance *I*, lung tissue energy dissipation *G*, and lung elastance *H *[14]. This approach has provided valuable insights in the pathophysiology of pulmonary functional impairment in various experimental models. For example, using FOT it was possible to show that the mechanism of exaggerated metacholin response in ovalbumin-challenged mice is primarily due to airway closure rather that smooth muscle contraction [41]. However, the utility of this methodology in the bleomycin model is less clear, since the studies so far have not described the temporal evolution of lung (dys)function in the model, nor have they examined the correlation of individual lung function parameters to morphological and biochemical indices. In addition, despite its theoretical advantages compared to classical PV-curves, which can be obtained with a variety of rodent ventilators, its superiority over the existing method has not been demonstrated experimentally in a manner convincing enough to justify the added cost. To address some of these issues, our study compared both methods to the biochemical and histological fibrosis indices of the bleomycin model.

In the early stages following bleomycin administration, we found, as expected, histological evidence of acute inflammation, which was associated with relatively mild changes in lung function, depending on the methodology used and the strain. In C57BL/6 mice, despite clear trends toward increased *H *and *G *by the FOT, statistical significance was not reached, possibly due to limited sample size. When lung mechanics were assessed using PV curves, however, the increase in PV-A was in fact statistically significant, implying that this method may have an advantage over the FOT in terms of sensitivity. At later time-points, inflammation is succeeded by fibrosis in the lungs of bleomycin-susceptible mice. Lung matrix expansion gradually leads to bridging of increasingly large areas of the lung with collagen fibres; beyond a certain threshold, a sharp rise in lung stiffness is thought to occur [38,39]. In functional terms, this process is expressed as a dramatic rise predominantly in *G*, *H *and to a lesser extent in PV-A. The observed correlation of *G *and *H *with fibrosis scores indicates that these functional perturbations are likely explained by the presence of matrix remodelling, with collagen deposition as its major feature. Intuitively, the rise in *H *could be attributed to increased tension in the alveolar walls during mechanical deformation, in conjunction with reduction of lung volume due to fibrosis. In the absence of lung volume measurements, the relative importance of these two factors cannot be quantified. The rise in *G *is less intuitive. Even though *G *reportedly represents inhomogeneity in airflow within the peripheral lung and has also been associated with increased peripheral airway resistance, [41] its meaning remains somewhat obscure [42] and *G *values are less frequently reported. In these studies, the increase in G may represent the tissue inhomogeneity inherent to fibrosis development. The ratio *G/H*, known as hysteresivity, was aimed at improving on some of the shortcomings of *H *or *G *alone. However, in these experiments due to the proportionate increase of *G *and *H*, hysteresivity did not change in any of the groups.

Lung mechanics assessment using quasi-static PV-curves yielded, to some extent, similar results with the data obtained by the FOT. PV-A increased significantly at the early time-point in C57/B6 mice and a further increase was recorded at the late time-point. However, this increase was of a lesser magnitude compared to the FOT data. Thus, correlation with fibrosis indices was weak at best but mostly absent. Taken together, these data indicate that the FOT may be more specific when it comes to detecting the presence of fibrosis in the mouse lung, whereas the PV-curve, although sensitive at recording lung dysfunction, may not allow the differentiation between the cause i.e. inflammation/edema vs. fibrosis.

The difference in susceptibility of C57B/6 and Balb/c mice to bleomycin-induced fibrosis is established in the literature [4,5,7,22-25,29,30,36] and thus Balb/c mice served as internal controls for sensitive C57B/6 mice. Studies comparing responses of these two strains to bleomycin report differences in gene expression [7], BAL cytokine profile [43] and ability for tissue repair [7]. Specifically, fibrosis-prone C57BL6 mice tend to have increased vascular protein leak [7], proteolytic enzyme activity [7], pro-inflammatory cytokine levels (MCP-1, TNF-α, IL-6) [7,43], TGF-β receptors [43], and apoptosis indices (caspase-8 and-9 activity). With respect to gene expression profile, Pottier et al. conducted a microarray analysis and found a set of 25 genes differentially expressed between the two strains, many of which were related to inflammatory response and tissue repair [7].

Predictably, the Balb/c strain did not develop fibrosis in response to bleomycin and had normal lung function by FOT. This finding may seem to contradict a recently published study reporting increases in *H *in bleomycin-exposed Balb/c mice [34]. However, in this paper a different protocol was used to induce lung fibrosis, since, in addition to bleomycin, intraperitoneal cyclophosphamide was also administered. Since collagen values are not reported in this study, it is not possible to interpret this discrepancy in findings. A surprising finding in our dataset, however, was that PV-A and slopes were significantly higher in bleomycin-compared to saline groups, indicating that despite lack of evidence for collagen accumulation, subtle mechanical changes take place in this strain as well. Furthermore, even though Balb/c mice developed similar degree of inflammation at the early time point as C57B/6 mice by morphological criteria, this was not reflected in lung function indices, for reasons yet to be determined.

One point of controversy regarding invasive lung function measurements in mice is whether these measurements should be performed in the presence or absence of PEEP. Use of PEEP helps to prevent alveolar collapse and reduction in lung volume during mechanical ventilation. However, we reasoned that this is more likely to occur in the presence of lung damage, including acute lung injury. While in our hands mice with unperturbed lungs tend to maintain stable values for *H *and *G *for several minutes, this is not the case with mice subjected to insults injurious to the lung. Therefore, avoiding PEEP may help unmask subtle functional abnormalities, which may be missed if PEEP is applied.

Our findings in bleomycin-treated mice are similar to the ones observed in humans with end-stage fibrotic lung disease. Published data from mechanically ventilated humans with end-stage fibrosis demonstrate that the disease is characterized by extensive scarring and reorganization of lung architecture, resulting in diminished lung volumes [44]. With loss of lung volume, the pressure-volume curve of the lung becomes contracted on its volume axis, thus reducing static and dynamic compliance. In this regard, the findings of the present study are in line with data from human subjects, a fact further validating the intratracheal bleomycin mouse model of lung fibrosis as an analogue to the human disease.

Although in our hands murine lung function measurements proved valuable indicators of bleomycin-induced lung fibrosis, some shortcomings of the present work and the method need to be pointed out. Firstly, procuring the equipment requires a financial investment. Secondly, we did not study lung function in other models of pulmonary fibrosis (eg, using silica or in genetic models), to further expand the possible uses of the method. Thirdly, it may be problematic to adjust tidal volume to body weight in the presence of fibrosis-induced weight loss, but this issue has not been resolved experimentally to our knowledge. Tidal volume in diseased animals may depend on a variety of factors, including functional dead space and carbon dioxide production, which are difficult to quantify in experimental mice. Thus, pending further experimental evidence, adjusting ventilation to body weight may currently be a reasonable option. And finally, since functional residual capacity was not measured in our study, some of the mechanical alterations observed following bleomycin challenge may be related to loss of lung volume, which would be expected in the presence of both acute lung injury and fibrosis.

The present work demonstrates that functional decline in the course of bleomycin injury is time-dependent and associated with the presence of fibrosis to a much greater extent than inflammation. We further show that for a more accurate assessment of lung function in rodent lung pathology models, complementary methodologies may be more informative than individual ones. Therefore, lung function measurements may serve as a valuable adjunct to pulmonary fibrosis research. Recent reports strongly advocate in favour of re-evaluating traditional histologic and biochemical tools for quantifying pulmonary fibrosis, since human trials for pulmonary fibrosis mostly use lung function parameters [1,25]. Based on our results, oscillatory measures of elastance and tissue damping are sensitive and reliable surrogates of fibrotic injury following bleomycin at later time-points, when fibrotic remodeling has had sufficient time to develop. These parameters reflect whole-lung visco-elastic behaviour and could serve as novel, user-independent end-points of lung fibrosis in future studies using this model.

## Conclusions

-Lung damage due to intratracheal administration of bleomycin can be divided into two stages, the inflammation stage and the fibrosis stage, with distinct functional profiles, at least when the forced oscillation technique is used.

-Lung elastance measured by the forced oscillation technique strongly correlates with soluble collagen content in lungs and histological indices of lung pathology.

-Quasi-static Pressure-Volume curves of the respiratory system sensitively detect lung dysfunction in the bleomycin model but seem to be less specific for fibrosis.

## Competing interests

The authors declare that they have no competing interests.

## Authors' contributions

EDM: Conducted experiments and analyses; wrote the paper; CM: conducted experiments; CT: Conducted experiments and analyses; AK: designed research; help with manuscript editing; IP: conducted analyses; SPK: conducted analyses; CR: help with manuscript editing; AA: help with manuscript editing; GTS: designed and conducted experiments and analyses; wrote the paper; NAM: designed and conducted experiments; wrote the paper.

All authors have read and approved the final manuscript

## Pre-publication history

The pre-publication history for this paper can be accessed here:

http://www.biomedcentral.com/1471-2466/11/33/prepub
